# Modeling human hepatic steatosis in pluripotent stem cell-derived hepatocytes

**DOI:** 10.1016/j.xpro.2021.100493

**Published:** 2021-04-21

**Authors:** Matthew C. Sinton, Jose Meseguer-Ripolles, Baltasar Lucendo-Villarin, Amanda J. Drake, David C. Hay

**Affiliations:** 1 University/BHF Centre for Cardiovascular Science, University of Edinburgh, The Queen's Medical Research Institute, Edinburgh BioQuarter, 47 Little France Crescent, Edinburgh EH16 4TJ, UK; 2 Centre for Regenerative Medicine, Institute for Regeneration and Repair, University of Edinburgh, Edinburgh BioQuarter, 5 Little France Crescent, Edinburgh EH16 4UU, UK

**Keywords:** Cell Differentiation, Stem Cells

## Abstract

This protocol describes the production of hepatocyte-like cells (HLCs) from human pluripotent stem cells and how to induce hepatic steatosis, a condition characterized by intracellular lipid accumulation. Following differentiation to an HLC phenotype, intracellular lipid accumulation is induced with a steatosis induction cocktail, allowing the user to examine the cellular processes that underpin hepatic steatosis. Furthermore, the renewable nature of our system, on a defined genetic background, permits in-depth mechanistic analysis, which may facilitate therapeutic target identification in the future.

For complete details on the use and execution of this protocol, please refer to Sinton et al. (2021).

## Highlights

• A detailed protocol to produce stem cell-derived hepatocyte-like cells
• Lactate, pyruvate, and octanoate treatment induces steatosis in hepatocyte-like cells
• A reliable tool for examining the processes that underpin hepatic steatosis

## Graphical abstract

**Figure:**
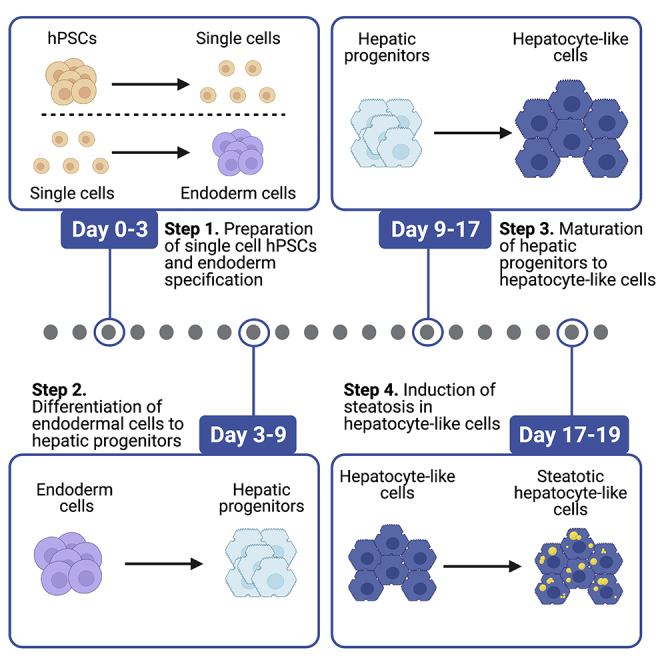

## Before you begin

### Coat culture plates with Laminin-521

**Timing: 30 min**

1. Thaw a 100 μg/mL stock of Laminin-521 (LN-521) for 16–18 h at 4°C.
2. Thawed LN-521 should be diluted in ice-cold 1X DPBS (containing Ca^2+^ and Mg^2+^), to generate a 5 μg/mL stock solution.
3. Add sufficient LN-521 to each well of a culture plate to coat. For a 6-well plate, add 1 mL of LN-521 to each well. For a 10 cm petri dish, add 5 mL of LN-521 to coat the plate.
4. After coating, incubate the plates at 4°C for 16–18 h on a flat surface. Alternatively, plates can be incubated in a 37°C/5% CO_2_ cell culture incubator for 2 h.
5. Plates should be sealed to prevent evaporation of LN-521, stored at 4°C, and used within 2 weeks.
6. Prior to use, allow the plates to equilibrate to 18°C–25°C by placing on a flat surface for 2 h. Alternatively, warm the plates in a 37°C/5% CO_2_ cell culture incubator for 30 min.

**CRITICAL:** It is critical that the LN-521 does not evaporate. Seal plates with parafilm to avoid evaporation. Following incubation, LN-521 coating, tip plates 45° and observe the growing surface. If the growing surface plastic can be seen when tipping, then coating has not been successful and must be repeated. If repeated coating is necessary, the addition of an extra 1 mL DPBS can reduce the chance of this occurring.

### Preparation of differentiation media, growth factors, and steatosis induction cocktail

**Timing: 4 h**

7. Stem cell media
  a. Add 100 ml mTeSR1™ 5X supplement to the 400 ml mTeSR1™ basal medium. Store for up to 1 month at 4°C.
8. Differentiation media
  a. To prepare endoderm differentiation medium, add 1% penicillin streptomycin and 1X B27 supplement to 500 mL RPMI 1640 medium. Store for up to 1 month at 4°C.
  b. To prepare hepatic progenitor differentiation medium, mix 400 ml knockout (KO)-DMEM, 100 ml KOSR Serum Replacement, 1% penicillin/streptomycin, 1% DMSO, 1% non-essential amino acids, 0.5% Glutamax and 0.2% β-mercaptoethanol. Store for up to 1 month at 4vC. This media formulation was previously optimized (Wang et al., 2017).
  c. To prepare hepatocyte maturation media, add 1% Glutamax, 1% penicillin streptomycin, 20 ng/mL hepatocyte growth factor, 10 ng/mL oncostatin M and 10 μM hydrocortisone 21-hemisuccinate sodium salt to HepatoZYME medium. Store for up to 1 month at 4°C.
9. Preparation of growth factors and other reagents
  a. Prepare a 1,000X stock of human activin A, by dissolving the lyophilized protein in sterile 0.2% bovine serum albumin (BSA; mixed with DPBS), giving a final concentration of 100 μg/mL. Prepare 30 μL aliquots and store at −20°C. Use at a 1:1000 dilution.
  b. Prepare a 1,000X stock of Wnt3A. Dissolve lyophilized mouse Wnt3A protein in sterile 0.2% BSA/DPBS, to a final concentration of 10 μg/mL. Prepare 30 μL aliquots and store at −20°C. Use at a 1:200 dilution.
  c. Prepare hydrocortisone 21-hemisuccinate sodium salt solution (HCC) by dissolving HCC in DPBS to a final concentration of 10 μM. Sterile filter the solution using a 22 μm filter and store in 5 mL aliquots at −20°C. Use at a 1:100 dilution.
  d. Prepare a 1,000X stock of human hepatocyte growth factor. Dissolve lyophilized HGF in sterile BSA/DPBS, to a final concentration of 10 μg/mL. Prepare 30 μL aliquots and store at −20°C. Use at a 1:1000 dilution.
  e. Prepare a 1,000X stock of oncostatin M. Dissolve lyophilized Oncostatin M (OSM) in sterile BSA/DPBS, to a final concentration of 20 μg/mL. Prepare 30 μL aliquots and store at −20°C. Use at a 1:1000 dilution.
  f. Prepare a 1,000x stock solution of Rho-associated kinase (ROCK) inhibitor Y-27632. Dissolve in sterile 0.2% BSA/DPBS to a final concentration of 10 mM. Prepare 10 μL aliquots and store at −20°C. Use at a 1:1000 dilution.
10. Prepare components of steatosis induction cocktail 24 h prior to use.
  a. Dissolve sodium L lactate in DPBS to a concentration of 1 M. Sterile filter the solution with a 22 μm filter. Store in 1 mL aliquots at −20°C for up to 1 week.
  b. Dissolve sodium pyruvate in DPBS to a concentration of 100 mM. Sterile filter the solution with a 22 μm filter. Store in 1 mL aliquots at −20°C for up to 1 week.
  c. Prepare octanoic acid to a 100 mM concentration in sterile water. Add lyophilized octanoic acid to sterile deionized water. Adjust pH to 7.0 – 7.9 by addition of 4 M NaOH solution with constant stirring. When solution becomes clear, store in 1 mL aliquots at −20°C for up to 1 week.

**CRITICAL:** Steatosis induction cocktail compounds stored for longer than 1 week become decreasingly effective and should be discarded.

11. Prepare steatosis induction cocktail on the day of use.
  a. Add lactate, pyruvate, and octanoic acid to HepatoZYME media, to a final concentration of 10mM, 1mM and 2mM, respectively. Sterile filter the solution prior to use.

## Key resources table

**Table:** 

REAGENT or RESOURCE	SOURCE	IDENTIFIER
**Chemicals, peptides, and recombinant proteins**		

Activin A	PeproTech	Cat#120-14E
Β-Mercaptoethanol	Gibco	Cat#31350-010
B-27™ Supplement (50X), minus vitamin A	Gibco	Cat#12587-010
BODIPY™ 493/503 (4,4-Difluoro-1,3,5,7,8-Pentamethyl-4-Bora-3a,4a-Diaza-s-Indacene)	Invitrogen	Cat#D3922
Bovine serum albumin	Sigma-Aldrich	Cat#A2058
^13^C_3_-Lactate	CK Isotopes	Cat#CLM-1579-05
Dimethyl sulfoxide	Thermo Fisher	Cat#D5879
DPBS (without Ca2+/Mg2+)	Thermo Fisher	Cat#14190144
GlutaMAX™ Supplement	Thermo Fisher	Cat#35050-038
HCS CellMask™ Red	Invitrogen	Cat#H32712
Hepatocyte growth factor	PeproTech	Cat#100-39
Human recombinant laminin 521	BioLamina	Cat#LN521-02
Hydrocortisone 21-hemisuccinate sodium salt	Sigma-Aldrich	Cat#H4881-1G
MEM Non-Essential Amino Acids Solution (100X)	Gibco	Cat#11140-035
Multidrop Combi Reagent Dispenser	Thermo Fisher	Cat#5840300
NucBlue Live ReadyProbes® Reagent	Molecular Probes	Cat#R37605
Octanoic acid	Sigma-Aldrich	Cat#C2875
Oncostatin M	PeproTech	Cat#300-10
Paraformaldehyde (4% wt/vol)	Electron Microscopy Sciences	Cat#15710-S
Penicillin-streptomycin (10,000 U/mL)	Gibco	Cat#15140-122
QIAzol	QIAGEN	Cat#79306
Rho-associated kinase (ROCK) inhibitor Y27632	Sigma-Aldrich	Cat#Y0503-1MG
RIPA Lysis and Extraction Buffer	Thermo Scientific	Cat#89900
Sodium L lactate	Sigma-Aldrich	Cat#L7022
Sodium pyruvate	Sigma-Aldrich	Cat#P5280
Triton X-100	Sigma-Aldrich	Cat#T8787
Universal ProbeLibrary – probe #7 (for use with ALB oligonucleotides)	Roche	Cat#4685059001
Universal ProbeLibrary – probe #27 (for use with HNF4A oligonucleotides)	Roche	Cat#4687574001
Universal ProbeLibrary – probe #69 (for use with NANOG oligonucleotides)	Roche	Cat#4688678001
Universal ProbeLibrary – probe #72 (for use with PLIN2 oligonucleotides)	Roche	Cat#4688953001
Universal ProbeLibrary – probe #1 (for use with PLIN4 oligonucleotides)	Roche	Cat#4684974001
Universal ProbeLibrary – probe #3 (for use with PLIN5 oligonucleotides)	Roche	Cat#4685008001
Universal ProbeLibrary – probe #87 (for use with TBP oligonucleotides)	Roche	Cat#4689127001

**Critical commercial assays**		

P450-Glo CYP3A4 Assay and Screening System	Promega	Cat#V8801

**Experimental models: cell lines**		

Human embryonic stem cell line H9	WiCell	Cat#WA09

**Oligonucleotides**		

ALB: Forward (GAACATCATGGATCAGAACAACA); Reverse (ATAGGGATTCCGGGAGTCAT)		N/A
HNF4A: Forward (AGCAACGGACAGATGTGTGA); Reverse (TCAGACCCTGAGCCACCT)		N/A
NANOG: Forward (ATGCCTCACACGGAGACTGT); Reverse (CAGGGCTGTCCTGAATAAGC)		N/A
PLIN2: Forward (TCAGCTCCATTCTACTGTTCACC); Reverse (CCTGAATTTTCTGATTGGCACT)		N/A
PLIN4: Forward (AGTTCCAAGCCAGGGACAC); Reverse (TGCTGGGCCTTTTCAATC)		N/A
PLIN5: Forward (TACAGTGCAGCCAAGGACAG); Reverse (CGCACACGCAGTTCTCAG)		N/A
TBP: Forward (GAACATCATGGATCAGAACAACA); Reverse (ATAGGGATTCCGGGAGTCAT)		N/A

**Software and algorithms**		

LightCycler® 480 Software	Roche	https://lifescience.roche.com/en_gb/products/lightcycler14301-480-software-version-15.html

**Other**		

96 Well Cell Culture Microplates, μClear®	Greiner Bio-One	Cat#655087
DPBS with Calcium and Magnesium	Thermo Fisher	Cat#14040133
Gentle cell dissociation reagent	STEMCELL Technologies	Cat#7174
GloMax explorer multiplex plate reader	Promega	Cat#GM3500
HepatoZYME-SFM	Gibco	Cat#17705-021
High-Capacity cDNA Reverse Transcriptase Kit	Applied Biosystems	Cat#4368814
Knockout DMEM	Gibco	Cat# 10829-018
KnockOut™ Serum Replacement (KO-SR)	Gibco	Cat#10828-028
LightCycler® 480	Roche	
Luna® Universal Probe qPCR Master Mix	New England Biolabs	Cat#M3004S
Monarch® Total RNA Miniprep Kit	New England Biolabs	Cat#T2010
mTeSR1 medium	STEMCELL Technologies	Cat#85850
Operetta CLS High-Content Analysis System	PerkinElmer	Part# HH16000000
PCR Plate, 384-well, standard	Thermo Scientific	Cat#AB1384
Pierce™ BCA Protein Assay Kit	Thermo Fisher	Cat#23225
Primer Thermal Cycler	Techne	N/A
RPMI 1640	Gibco	Cat#11875-093
TURBO DNA-free™ Kit	Ambion	Cat#
White plates for CYP assays	Greiner Bio-One	Cat#655075

## Step-by-step method details

### Passaging human pluripotent stem cells (hPSCs) in preparation for differentiation

**Timing: 1 day**

**Figure 1:**
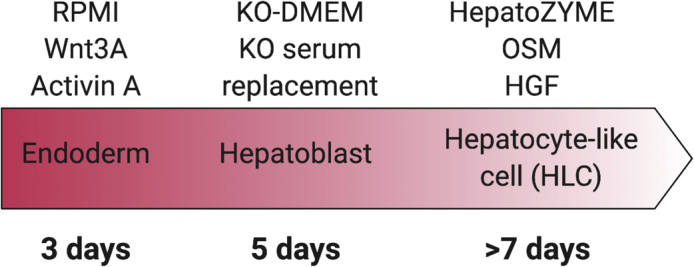
Schematic of differentiation processes and treatments required at each timepoint

This step describes the preparation of hPSCs for differentiation to hepatocyte-like cells (HLCs) (Figure 1). The protocol requires that hPSCs are at a confluence of 75%–80%. For passaging, hPSCs must be in a single cell suspension. This protocol was adapted from (Lyall et al., 2018; Meseguer-Ripolles et al., 2018; Wang et al., 2017).

1. When hPSCs are at 75%–80% confluence, aspirate media.
2. Wash each well with 18°C–25°C 1X DPBS (without Ca2+/Mg2+) and then aspirate.
3. Add 1 mL of Gentle Cell Dissociation Reagent (STEMCELL Technologies™) to each well and incubate cells at 37°C/5% CO_2_, for 5–8 min, to promote dissociation of cells from the growing surface.
4. Whilst cells are dissociating, prepare the Laminin-521-coated plates. Carefully aspirate Laminin-521 from the well, without disturbing the coating on the growing surface.

**CRITICAL:** Do not allow the Laminin-521 to dry out at any point. If the Laminin-521 pools and exposes a dry plastic growing surface, then coating has not been successful and must be repeated prior to starting the differentiation process.

5. Immediately after aspirating, add 1 mL of supplemented mTeSR1™, pre-warmed to 37°C, containing 10 μM Rho-associated kinase (ROCK) inhibitor Y-27632 to each well of a 6-well plate. This is half of the media that will be added to each well.

***Note:*** ROCK inhibitor Y-27632 is used to enhance hPSC attachment and survival in single cell suspensions.

6. Incubate the LN-521 coated plates, with ROCK-supplemented media, at 37°C/5% CO_2_ until the hPSCs are dissociated and ready for plating.
7. To determine whether hPSCs have formed a single cell suspension, observe the plates under a standard light microscope. If the cells are not ready, increase the incubation step, to a maximum of 10 min. Cells will start to look round when their edges are detaching from the growing surface.
8. Aspirate the Gentle Cell Dissociation Reagent and immediately add 1 mL of pre-warmed supplemented mTeSR1™ with 10 μM ROCK inhibitor Y-27632 to each well.
9. Detach cells from the growing surface using a cell scraper.
10. Collect the media, containing suspended hPSCs, into a 15 mL Falcon tube and centrifuge at 200 × *g* for 5 min, at 18°C–25°C.
11. Aspirate the media without disturbing the cell pellet and then tap the tube to ensure that there are no clumps.
12. To the Falcon tube, add 10 mL of supplemented mTeSR1™ with 10 μM ROCK inhibitor Y-27632. Slowly pipette up and down to resuspend the cell pellet. Count the cells using an automatic cell counter and use Trypan Blue to exclude dead cells. Count cells three times and calculate the mean number of cells.

***Note:*** Manual counting is not recommended as it introduces user-to-user variation

13. Seed the cells at a density of 5.0 × 104 cells per cm2 growing surface. Seeding density may require optimization for different well sizes or shapes.
14. Place the plate on a flat surface and gently shake the plate up and down, then left to right, for 10 times each.
15. Incubate the hPSCs at 37°C/5% CO2 for 24 h before starting the differentiation protocol.

### Hepatocyte-like cell differentiation

**Timing: 17 days**

This protocol describes how to differentiate hPSCs to hepatocyte-like cells (HLCs) and generates homogeneous populations. This protocol below was previously described (Wang et al., 2017).

**Figure 2:**
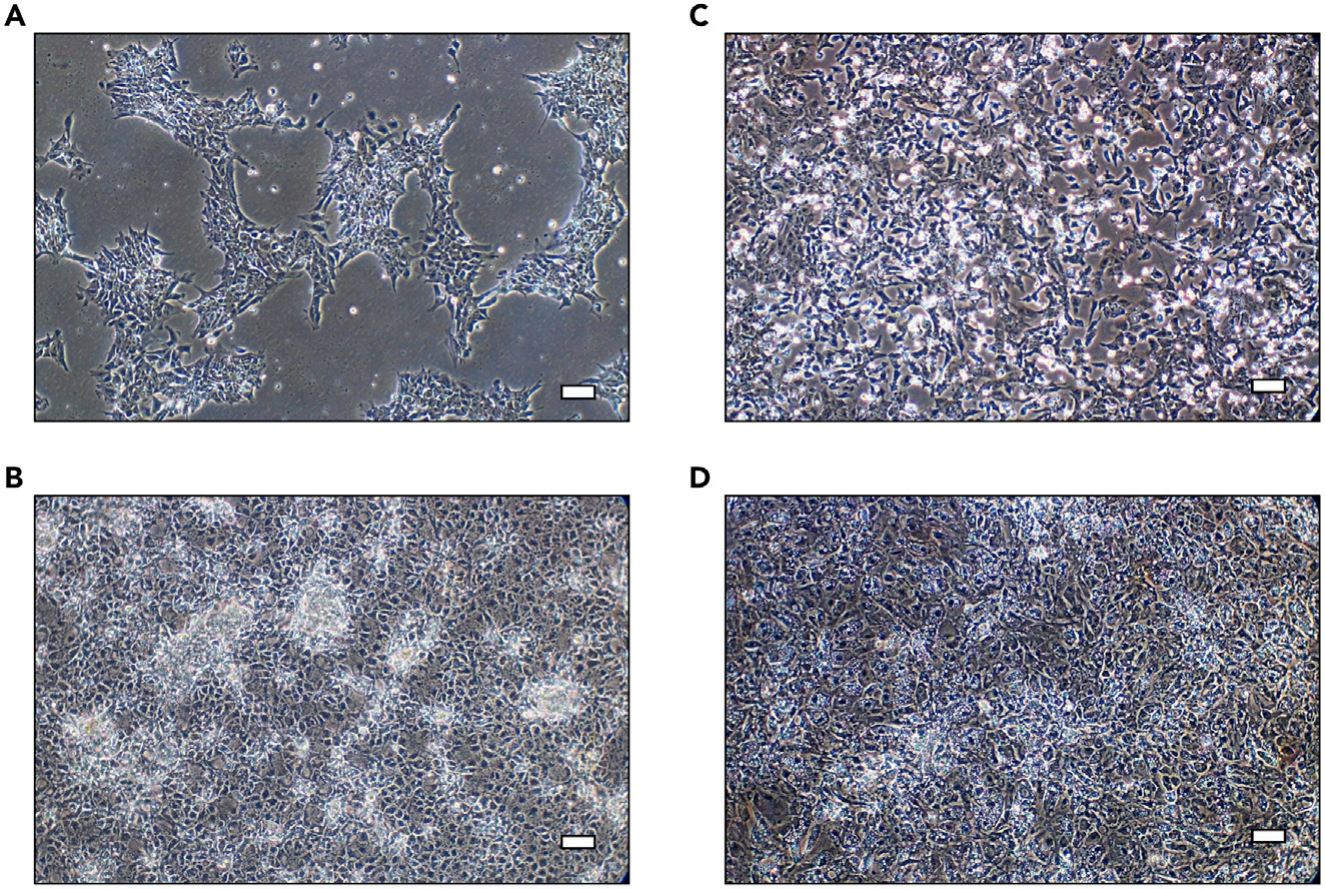
Differentiation of H9 cells leads to associated changes in cell morphology Representative images of cell morphology during the differentiation process. (A) H9 cells approximately 24 h after seeding onto Laminin-521 coated plates and reaching 40% confluence. (B) H9 cells on day 3, at the endoderm stage. (C) H9 cells on day 9, following induction of hepatoblast differentiation. (D) H9 cells at day 17, following maturation to HLCs. Scale bar, 25 μm. Adapted from Sinton et al 2021, iScience, https://doi.org/10.1016/j.isci.2020.101931
17. Aspirate the supplemented mTeSR1™ and replace with 2 mL RPMI 1640 supplemented with 100 ng/mL Activin A and 50 ng/mL Wnt3A. Incubate the cells at 37°C/5% CO_2_ and refresh the media every 24 h for 3 days.
18. On day 3, H9 cells should achieve an endodermal morphology (Figure 2B). Aspirate endoderm differentiation medium and replace with hepatic progenitor differentiation medium. Incubate the cells at 37°C/5% CO_2_ and refresh the media every 48 h for 5 days.
19. On day 9, H9 cells should achieve a hepatoblast morphology (Figure 2C). Aspirate the hepatic progenitor differentiation medium and replace with hepatocyte maturation medium, supplemented with 10 ng/mL hepatocyte growth factor and 20 ng/mL oncostatin M. Incubate the cells at 37°C/5% CO_2_ and refresh the media every 48 h for 9 days.
20. On day 17, H9 cells should achieve a hepatocyte morphology (Figure 2D). HLCs are now mature and ready to be used in downstream assays.
21. To determine that cells are functioning as mature hepatocytes, measure CYP3A4 activity on day 17, using the P450-Glo™ assay (Promega), as per the manufacturer’s instructions (https://www.promega.co.uk/products/cell-health-assays/adme-assays/p450-glo-cyp3a4-assay-and-screening-system/?catNum=V9001#protocols). Results should be normalized to protein content of the well analyzed. Measure protein using the Pierce™ BCA Protein Assay Kit as per the manufacturer’s instructions (https://www.thermofisher.com/order/catalog/product/23225#/23225).

***Note:*** At days 0, 3, 9 and 17, collect cells to measure mRNA of the pluripotency marker NANOG, and the hepatocyte markers HNF4A and ALB (Figure 3). This enables the tracking of the changing cell phenotype throughout the differentiation process.

16. Once hPSCs reach 40% confluence (Figure 2A), normally 1 day after seeding, begin the differentiation process. This timing is variable and it can take between 18 and 24 h for cells to reach 40% confluence if cell counts are not accurate.

**Figure 3:**
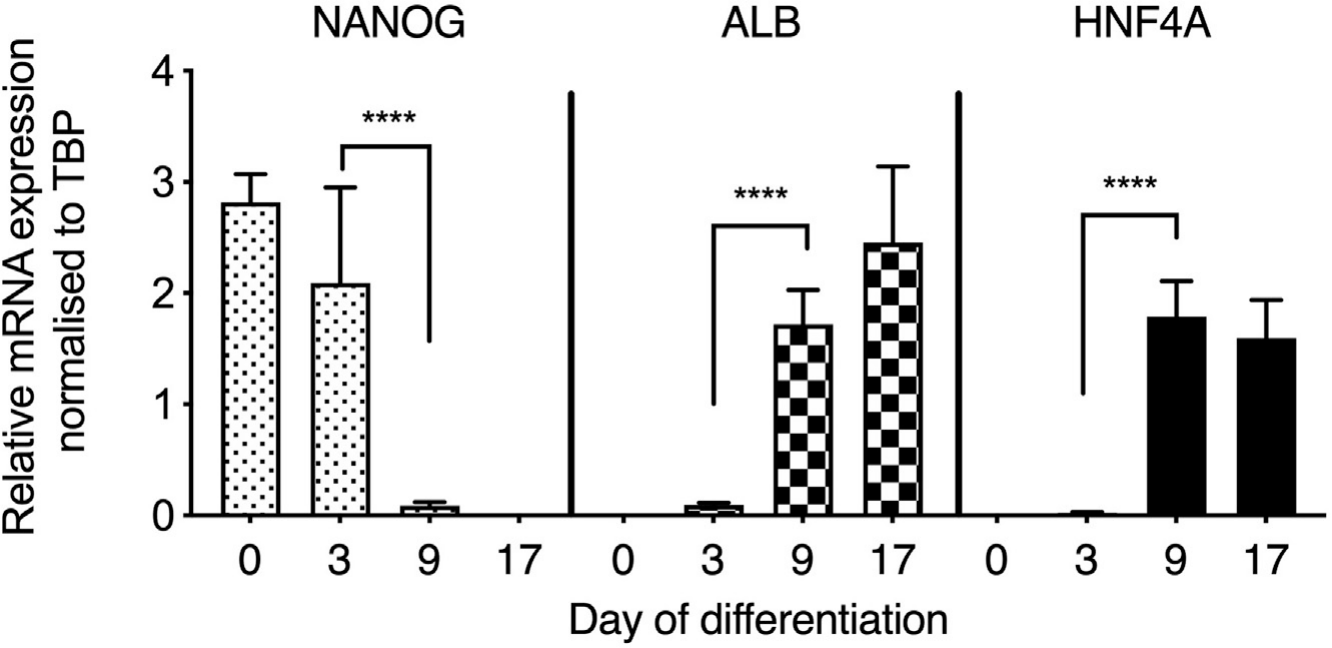
Differentiation process leads to loss of pluripotency and gain of hepatocyte maturation markers Temporal expression of pluripotency marker (NANOG) and hepatocyte markers (ALB and HNF4A) during differentiation of hPSCs to HLCs (data are expressed as mean± SD; n = 8). Adapted from Sinton et al 2021, iScience, https://doi.org/10.1016/j.isci.2020.101931

### Induction of and verification of steatosis

**Timing: [2 days]**

This step leads to the accumulation of intracellular lipids within the HLCs, and mimics human hepatic steatosis. This protocol was adapted from (Lyall et al., 2018).

22. On day 17 of HLC differentiation, aspirate the hepatocyte maturation media. Replace this with hepatocyte maturation media supplemented with sodium L-lactate (10 mM), sodium pyruvate (1 mM) and octanoic acid (2 mM). Incubate the cells at 37°C/5% CO_2_ for 48 h.

***Note:*** Following induction of steatosis, cells from a single well can be used for either imaging or mRNA collection, but the same well cannot be used for both purposes.

23. For microscopy analysis of lipid accumulation, aspirate cell media and replace with 4% (weight/volume) paraformaldehyde in DPBS. Incubate at 18°C–25°C for 15 min.
24. Aspirate the paraformaldehyde and replace with DPBS containing 0.1% Triton X-100 for 15 min at 18°C–25°C, to permeabilize the cells.
25. Stain the cells with 2 μL/10 mL HCS CellMask™ Red, 2 drops/mL NucBlue Live ReadyProbes® Reagent, and 1:1000 BODIPY™ 493/503. Following incubation with these stains, incubate in the dark, at 18°C–25°C, for 30 min.
26. Following staining, use fluorescence microscopy to assess intracellular lipid accumulation. For this purpose, we used an Operetta CLS High-Content Analysis System.
27. To further confirm lipid accumulation, mRNA of markers for lipid droplets, perilipin 2 (PLIN2), PLIN4 and PLIN5 can be measured.
28. To purify mRNA from HLCs, wash cells twice with DPBS. Incubate the cells with 1 mL QIAzol for 5 min at 18°C–25°C. Then purify mRNA using the Monarch® Total RNA Miniprep Kit as per the manufacturer’s instructions (https://www.neb.uk.com/products/neb-catalogue/nucleic-acid-purification/monarch-lt;sup-gt;-amp;reg;-lt;-sup-gt;-total-rna-miniprep-kit).
29. Reverse transcribe RNA using the TURBO DNA-free™ Kit according to the manufacturer’s instructions (https://www.thermofisher.com/order/catalog/product/AM1907#/AM1907).
30. Measure mRNA using the Luna® Universal Probe qPCR Master Mix, as per the manufacturer’s instructions (https://international.neb.com/products/m3004-luna-universal-probe-qpcr-master-mix#Protocols,%20Manuals%20&%20Usage) and the conditions outlined in Table 1.

**Table 1:** Cycling conditions for qPCR measurements of mRNA

qPCR cycling conditions			
Steps	Temperature	Time	Cycles
Preincubate	95°C	5 min	1
Denaturation	95°C	10 sec	40 cycles
Annealing/extension	60°C	30 sec	
Cool	40°C	30 sec	1
Hold	4°C	Infinite	1

***Note:*** Measurements of lipid marker mRNA should be used in tandem with microscopy to give confidence that steatosis is developing in HLCs treated with lactate, pyruvate, and octanoic acid.

## Expected outcomes

**Figure 4:**
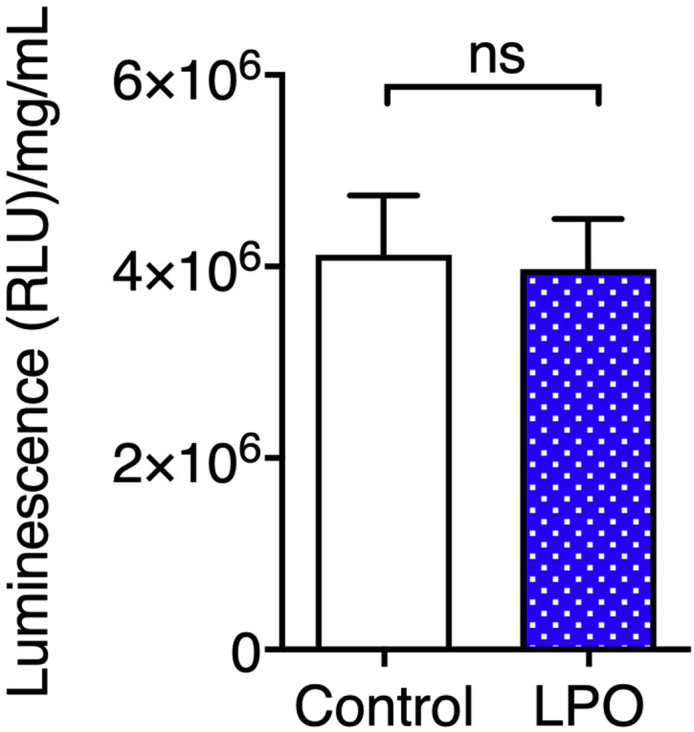
Maturation of HLCs is associated with functional activity typically associated with human hepatocytes Cytochrome P450 3A4 activity in HLCs following differentiation with or without the lactate, pyruvate, octanoic acid (LPO) cocktail (data are expressed as mean± SD; *n* = 8). Adapted from Sinton et al 2021, iScience, https://doi.org/10.1016/j.isci.2020.101931

**Figure 5:**
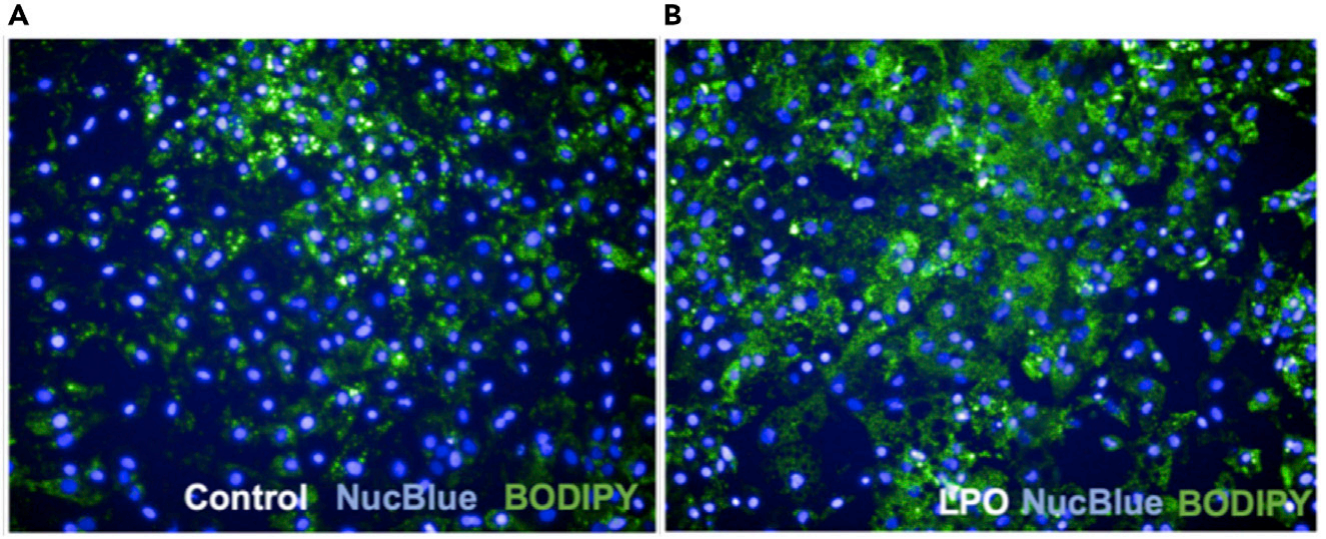
Treatment of HLCs with steatosis induction cocktail leads to intracellular lipid accumulation Representative images of lipid accumulation in control (A) and LPO-treated (B) groups; 10x magnification. Hoechst dye (NucBlue) and BODIPY were used to stain nuclei or neutral lipids, respectively. Adapted from Sinton et al 2021, iScience, https://doi.org/10.1016/j.isci.2020.101931

**Figure 6:**
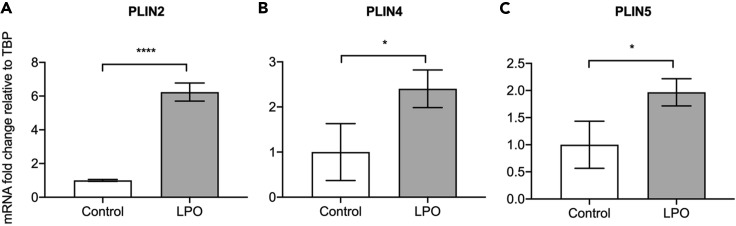
Accumulation of intracellular lipids in HLCs is associated with increased lipid droplet markers Expression of PLIN2 (A), PLIN4 (B) and PLIN5 (C) was measured. For each group *n* = 3 biological replicates. Data were analyzed using a two-tailed Student t-test and expressed as mean ± SD. ∗p<0.05, ∗∗∗∗p<0.0001. Adapted from Sinton et al 2021, iScience, https://doi.org/10.1016/j.isci.2020.101931.

This protocol generates stem cell-derived hepatocyte-like cells, which are phenotypically similar to mature human hepatocytes. Figure 3 demonstrates that following differentiation, cells acquire expression of markers typically associated with hepatocytes, which they lack in the pluripotent state. HNF4A activity is understood to be crucial for hepatic progenitor specification (Wang et al., 2019) and, therefore, it is essential to confirm that cells are expressing this marker prior to hepatocyte maturation. Figure 4 highlights that these cells also acquire functional activity that is representative of cytochrome activity associated with primary hepatocytes. Following treatment with lactate, pyruvate, and octanoic acid, HLCs accumulate greater volumes of intracellular lipid, as demonstrated by BODIPY staining in Figure 5. This is accompanied by an increase in expression of markers associated with lipid droplet biogenesis – PLIN2, PLIN4 and PLIN5, highlighted in Figure 6. Recently developed in silico tools enable mathematical modeling of oxygen gradients within liver tissue, providing insight into how these gradients will impact on cell phenotype (Leedale et al., 2021). This platform can be scaled and provides an excellent tool to study liver biology and disease (Lucendo-Villarin et al., 2020a, 2020b; Sinton et al., 2021)*.*

## Limitations

Although HLCs are morphologically and functionally similar to hepatocytes, they do not necessarily recapitulate the entire transcriptome of human hepatocytes (Godoy et al., 2015). Whilst LPO-treated HLCs express a substantial number of steatosis-related genes (Sinton et al., 2021), it is unclear how expression patters are impacted by the lack of co-culture with other liver-resident cells, such as endothelial, stellate or Kupffer cells, but this system still provides invaluable insights into the response of hepatocytes to steatosis.

## Troubleshooting

### Problem 1

Following coating of tissue culture plastic with Laminin-521, the coating does not fully cover the well, exposing the growing surface.

### Potential solution

Coating of plates with Laminin-521 is vital prior to cell seeding. Check that the correct concentration of Laminin-521 was used and that the plates were incubated for the correct length of time. Repeat the coating process and if the problem persists, increase the volume of Laminin-521 in the well and ensure that plates are being stored on a completely flat surface.

### Problem 2

Following seeding of H9s onto wells coated with Laminin-521, there are high levels of cells death, leading to sparse seeding of the well.

### Potential solution

When preparing a single cell suspension of H9 cells, media must contain ROCK inhibitor Y-27632 to prevent excessive cell death. Prior to addition of endoderm differentiation medium, H9 cells that are seeded for differentiation should be incubated in mTeSR1™ basal medium containing ROCK inhibitor Y-27632.

### Problem 3

When seeding single cells prior to differentiation, cells may not be distributed homogeneously, leading to heterogeneous patterns of differentiation within the well. If this occurs, by day 9 of the differentiation process, cells may not reach confluence and morphology will not appear consistent across the well.

### Potential solution

Attachment of the pluripotent stem cells to Laminin-521-coated plates occurs rapidly. To ensure that cells are distributed evenly across the growing surface of the well or plate, gently agitate the plate up and down, and side-to-side, at least 10 times prior to transfer to the incubator.

### Problem 4

Differentiation of H9 cells to the endoderm, hepatoblast or HLC specification are unsuccessful as determined by qPCR analysis of pluripotency of hepatocyte markers.

### Potential solution

Check that the components of each differentiation media have been prepared to the correct concentration and stored under the correct conditions.

### Problem 5

Induction of steatosis may be unsuccessful, as determined by BODIPY staining and immunofluorescence.

### Potential solution

Components of the steatosis-induction cocktail have a limited shelf life. It is strongly recommended that storage conditions are checked and that fresh solutions are prepared if cells are not developing steatosis following 48 h of incubation with this cocktail.

## Resource availability

### Lead contact

Further information and requests for resources and reagents should be directed to and will be fulfilled by the lead contact, David C. Hay (davehay@talktalk.net).

### Materials availability

Further information and requests for resources and reagents should be directed to and will be fulfilled by the lead contact.

### Data and code availability

Datasets will be made available upon request to the lead contact.
